# Towards Compensation for Servo-Control Defects in Coordinate Measuring Machines (CMMs)

**DOI:** 10.3390/s25133956

**Published:** 2025-06-25

**Authors:** Jean-François Manlay, Abdérafi Charki, Anthony Delamarre

**Affiliations:** 1 CEA/DAM/Gramat, Route de Reilhac, 46500 Gramat, France; 2 University of Angers–LARIS, 62 Avenue Notre Dame du Lac, 49000 Angers, France; abderafi.charki@univ-angers.fr (A.C.); anthony.delamarre@univ-angers.fr (A.D.)

**Keywords:** coordinate measuring machine, servo-control, runout, circularity, roundness

## Abstract

**Highlights:**

This study focuses on the effects of servo-control while measuring circles on warped surfaces with CMMs. It describes the methods for identifying the various parameters that affect the measurement. It offers innovative solutions to overcome positioning accuracy problems and to improve results such as circularity or runout measurements. Two practical solutions are suggested to avoid the servo-control defects.

**What are the main findings?**

- In some cases, constructing a circle is a better solution than measuring it directly.
- Our study showed that servo-control can have a significant impact on specific CMM measurements.
- Two methods are hereby proposed in order to circumvent the effect of servo-control.

**What are the implications of the main findings?**

- They allow for the accuracy of some results on circles to be increased.
- They allow for standard specifications to be met.

**Abstract:**

Coordinate measuring machines (CMMs) are increasingly used in manufacturing, mechanical engineering, and wherever special geometries need to be measured with the utmost precision. CMMs are very important in various fields including the automotive, aerospace, and military industries. For certain specific tasks, such as measuring roundness or contour, they are not as accurate as specialized measuring machines, for instance, roundness measuring machines, especially if the circle is to be measured on an oblique surface. The CMM servo loop is not as accurate as the CMM readings, as it leads to differences between the theoretical target coordinates of a point and the actual coordinates obtained. On a conical surface, for example, where height and radius are linked, these differences are the cause of errors on circle dimensions. In this case, it is necessary to construct the feature instead of measuring it directly. This article proposes innovative methods for performing specific tasks on a CMM and for taking faults due to servo-control into account. The results show significant improvements for standard parts or skewed surfaces.

## 1. Introduction

A coordinate measuring machine (CMM) is a measuring instrument that uses point acquisition to create geometric elements in order to verify the conformity of a manufactured part to its nominal definition in terms of both dimensions and shapes. More precisely, point acquisition means acquiring the three-dimensional coordinates of points in a reference frame linked to the part or machine itself. This ability to measure different types of characteristics with a single instrument makes these machines very interesting, though not perfect.

Whatever the acquisition technology is, every point coordinate is subject to measurement uncertainty, which is consequently propagated to the creation of the associated geometric elements, in terms of dimension, shape, orientation, and position.

Coordinate measuring machines (CMMs) are used for their accuracy, within an acceptance tolerance, and are verified using calibrated standards [1]. However, the methods used to carry out the measurements do not allow us to assess the uncertainty of measurement on a conventional part [2].

The study of the influence of CMM servo-control on measurements is rarely described in depth in the literature. A great deal of research has been carried out since the 1970s to improve the servo-control of machine tools [3,4], but the work on CMM servo-control has not been sufficiently developed. Coorevits [5] addresses the problem of different probing errors, which are also presented by Bouaziz [6]. They distinguish between the error on the approach vector and the tangential one on measurements of points, with the latter fault being described as a servo-control defect in [6], which is negligible for large curvature and fine roughness. Servo influence on features other than points is not treated.

In a few cases, particularly when measuring circles on axisymmetrical skewed surfaces, servo-control errors lead to measurement faults that misinterpret the actual geometry of the part.

Servo-control defines the function between CMM movements and the actual position read on the measuring scales. Machine movements between two measured points can be broken down into three phases: retraction, displacement, and approach. These movements are generated at different speeds and accelerations depending on the distance between points. The speed decreases to measuring speed when the pre-hit distance is reached [5]. In continuous scanning mode, speed and acceleration are set in the measurement parameters. This real-time adjustment is not as precise as the readout position, so there may be a difference of a few micrometers between the theoretical target and the actual target.

The servo-control neither affects the acceptance test nor the most conventional surface dimensions (planes, cylinders, etc.), especially when the associated element is constructed from center ball hits, before ball compensation. Despite being measured on a three-dimensional cylinder, a circle is a plane figure. The CMM servo-control does not permit the machine to move the probe in a perfect plane. Parasitic displacements parallel to the cylinder axis do not affect circle parameters in this case.

However, some dimensions cannot be accurately measured on a CMM without certain precautions being taken. This is the case for circularity and radial runout when they are located on conical or deformed surfaces, or any axisymmetric surface if it is not cylindrical. On these types of surfaces, the drive cannot control the probing system on a perfect plane [7], so the measurement takes radial deviations due to axial discrepancy into account. Section 3.2.2 provides a comprehensive overview of the impact of the servo-control fault between circles measured on cylinders or on cones.

Numerous research studies have been conducted on roundness and circularity, examining the effect of various parameters, except for servo-control. Nemedi [8] compares the results obtained with different measuring systems, including CMMs and roundness measuring machines. Stepien [9] shows the influence of scanning speed and probing direction on the roundness result. Drbùla [10] also explains the effect of probing direction on conical measurements.

Gapinski [11,12] studies the evolution of results as a function of the angular sector measured by applying different algorithms. Gass [13] presents the processing of the same measurements by different algorithms, such as least squares, Chebyshev, circumscribed minimum, or inscribed maximum. Samuel [14] and Gadelmawla [15] present different treatments of CMM data to assess circularity. Studies on runout are less numerous than those on circularity, and they are often in the field of gear measurements. In [16], Guenther presents a method for measuring pitch. Lin [17] studies the geometrical defects of the CMM on gear measurements, and Tao [18] presents a method for measuring gear teeth. Without directly naming this as an effect of servo-control, Mazur [19,20] describes the differences in positioning between the target and the acquired points on different types of surfaces. He also describes the absence in the ISO 10 360 standards [21] of parameters such as positioning accuracy and repeatability, which are found in robot standards [22].

Examples of uncertainty evaluation are presented for circularity by Jalid [23], and another for taper by Wang [24]. Among all the publications relating to these studies, those presenting data sets allowing the servo-control error to be calculated are rare [19,20]. Some articles present results in two dimensions only [11,12,14,15], which does not allow the servo-control error to be identified. In these studies, its effect has no influence on the results, as the measured features are not distorted, except for certain gear or cone measurements. NIST (the National Institute of Standards and Technology of the United States) uses different data sets to test the algorithms [25]. Sets that mimic CMM measurements can be used to highlight the servo-control fault, even if this is not the desired objective.

A servo-control error can be estimated by the distance between the theoretical target and the measured target. It results from the interpretation of real-time scale readings and the positioning command sent by the controller. This fault depends on various parameters, such as travel speed, probe offsets, electronic configuration, and the number of axes required for travel. It has no influence on most shape dimensions (flatness, cylindricity, surface profile, etc.), as the positioning deviation is not very significant compared to the distance between points. Nevertheless, it may affect measurement repeatability in the case of surfaces with high roughness measured with a small probe diameter, which is not recommended either [26].

To identify servo-control faults, it is necessary to access the actual coordinates of the measured points via the software. However, access to these data is not always direct.

The use of a calibrated ring gauge makes it possible to pinpoint different aspects of a servo-control fault while retaining the diameter and circularity measurement references.

In this article, we suggest a way of characterizing the effect of servo-control on the measurement of the coordinates of a point, a circle, and a deformed surface, respectively. Two solutions are presented to avoid servo-control defects. The proposed method is demonstrated in the case of a calibration sphere and for the measurement of a torus. This study does not deal with probe triggering (TTP), which itself presents too many deviations in circularity measurements due to the lobing of the probe, even if some improvements have been described in [27,28]. Some TTP models are equipped with three spring-loaded contacts. Depending on the probing direction, one, two, or three contacts will open. The probing force is, therefore, linked to the force of one, two, or three springs, so the measurement of a circle may present a so-called “three-lobed” defect in the shape of a triangle. We limit the scope of our study to scanning heads, which avoid the three-lobed circles of the TTPs [26].

Section 2 of this article describes the proposed innovative methodology. Before concluding, Section 3 deals with the measurement results obtained from several geometric configurations.

The aim of this paper is to help metrologists characterize certain hidden parameters of their CMMs, especially the servo-control, and then reduce the uncertainties of their results.

## 2. Materials and Methods

In this section, a servo-control defect is assumed to exist, whatever its value or its cause. As described in the Introduction, the servo-control is unable to ensure the same precision in CMM moves as in the CMM readings. This results in a number of defects, which are presented in Section 3. Warped surfaces represent features such as cones, spheres, toruses, or any axisymmetric surface other than a cylinder. These surfaces are supposed to be sensitive to servo-control.

All the equations presented below can be translated into certain CMM software, or used as post-processing.

Both of the methods presented necessarily begin by defining the datum system in order to create the right direction for measurements and basic dimensions.

### 2.1. Defining Servo-Control

The servo-control of a CNC (CNC: Computer Numerical Control) machine describes the relationship between a movement command sent to the controller and the actual movement of the machine. The difference between the theoretical target and the actual coordinates should be small, but it is not zero. Most features are unaffected by this function, with the exception of point coordinates and circles measured on deformed surfaces. For example, on a conical surface, an axial deviation creates a radial deviation. In the case of a cylinder, an axial deviation does not affect the radius measurement. This axial dispersion can be observed while measuring a circle, without affecting the results. This measurement may be used to define two defects, such as the thickness of the point cloud and the angle between the actual plane displacement of the probe and its theoretical path. Those parameters are developed in Section 3.

### 2.2. First Method

**Figure 1:**
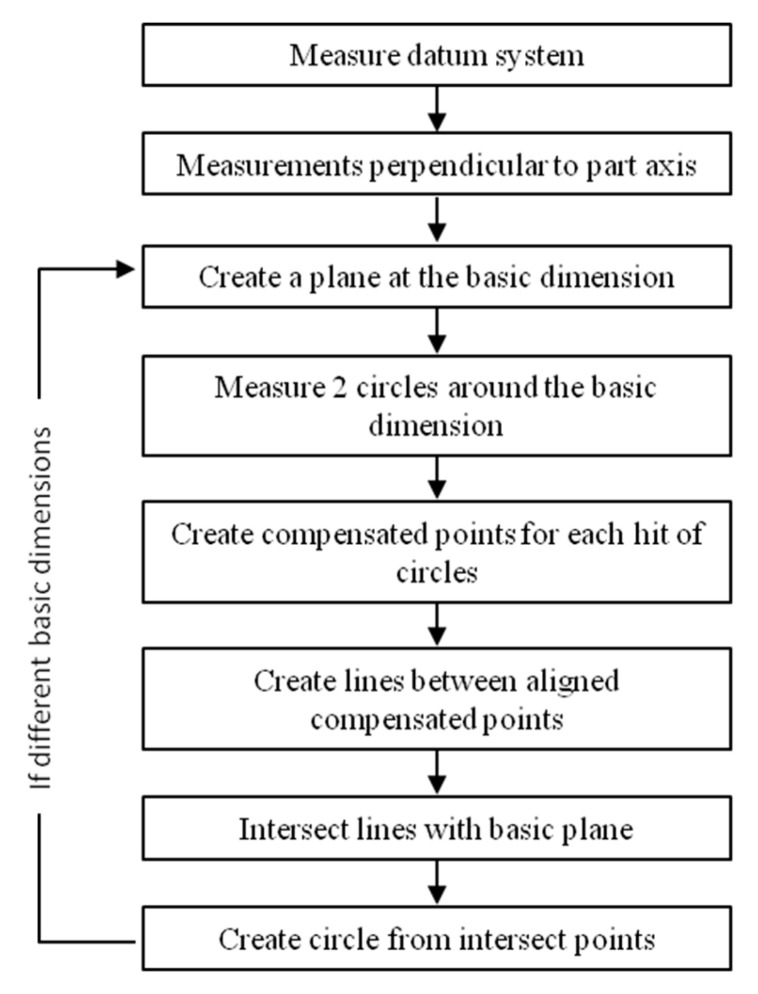
Sequence of operations to perform the first method.

To avoid the servo-control fault, which affects the results of circles measured on warped surfaces, the first solution consists of constructing the circle from two measured circles instead of one (Figure 1).

**Figure 2:**
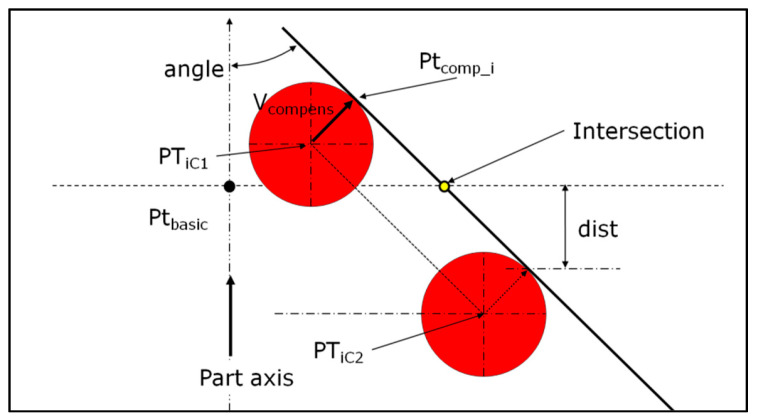
Constructing a circle at the correct height from two measured circles using the center ball of the probe and compensation vectors.

The method requires the measurement of circles with the same number of hits in the same direction, if possible. Both circles (*C*_1_ and *C*_2_) must be close together, from a few hundredths to a few tenths of a millimeter around the basic dimension, with the distance between them depending on the surface curvature (Figure 2). The closer the circles, the greater the accuracy of the result. However, the defect characterization described in Section 3 enables the shortest distance to be respected between circles to be defined. This ensures that all point-to-point vectors are pointing in the same direction.

The calculation uses center ball coordinates for each hit (Figure 2) following these different steps:

- The construction of unit vectors between center ball coordinates $\vec{U_{{PT}_{i}}}$, then a radial vector $\vec{R_{i}}$ perpendicular to it and to the part’s axis.

(1) $$\vec{U_{{PT}_{i}}}=\frac{\vec{{PT}_{iC2}{PT}_{iC1}}}{\left\|\vec{{PT}_{iC2}{PT}_{iC1}}\right\|}$$

(2) $$\vec{R_{i}}=\frac{\vec{U_{{PT}_{i}}}\wedge \vec{axis}}{\left\|\vec{U_{{PT}_{i}}}\wedge \vec{axis}\right\|}$$

- The compensation vector is perpendicular to $\vec{U_{{PT}_{i}}}$ and $\vec{R_{i}}$.

(3) $$\vec{V_{compens}}=\pm \vec{U_{{PT}_{i}}}\wedge \vec{R_{i}}$$

*V_compens_* is a unit vector by definition of the cross product between two unit perpendicular vectors. The dot product between the compensation vector and the approach vector must be positive. This allows for the compensation of the ball radius on the right side.

- The compensated points are as follows.

(4) $$\vec{{OPT}_{comp\_i\_2}}=\vec{{OPT}_{iC2}}+R_{ball}\vec{.V_{compens}}$$

- The distance (*dist*) between a compensated point and the basic plane is given by (5).

(5) $$dist=\vec{{PT}_{comp\_i\_2}{Pt}_{basic}}.\vec{axis}$$

- The angle (*angle*) between the vector constructed from center balls and the basic plane is given as follows.

(6) $$angle=Acos\left(\vec{U_{{PT}_{i}}}.\vec{axis}\right)$$

- The coordinates of the constructed point (${PT}_{intersect\_i}$) are determined with the following expression.

(7) $$\vec{{OPT}_{intersect\_i}}=\vec{{OPT}_{comp\_i\_2}}\pm \frac{dist}{cos(angle)}\left(\vec{U_{{PT}_{i}}}\right)$$

In (7), *cos*(*angle*) can be replaced by $\vec{U_{{PT}_{i}}}.\vec{axis}$ because the dot product is calculated between unit vectors.

Then, the circle can be constructed from all the constructed points, which are coplanar to the basic plane. If the circles are measured using the normal vector of the feature, then the coordinates of the contact point can be used directly.

In the event of continuous scanning, the number of hits of both circles could vary if the software does not allow it to be set. Should the parameter setting be scan density, a straightforward solution would be to calculate the diameter ratio (larger/smaller). Multiplying the density of the scan conducted on the larger diameter by the ratio should provide an equal, or very close to equal, number of hits to the scan on the smaller diameter. The construction of intersected points can be time-consuming in online treatment, depending on the software, due to the large number of hits. It may be worthwhile to consider creating these points in the post-treatment phase.

**Figure 3:**
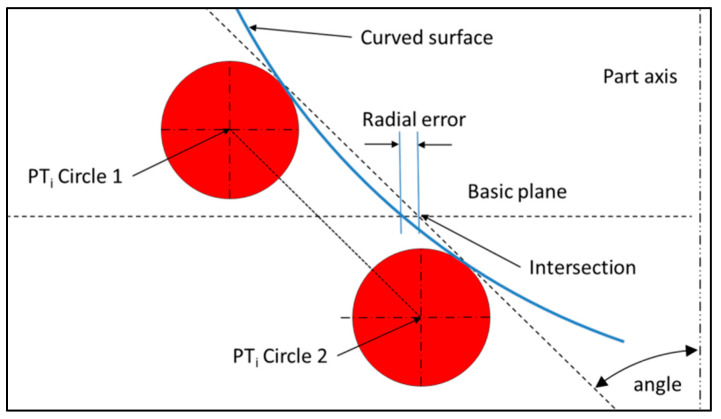
Radial error while dimensioning the circle’s radius because of the local curvature.

This method generates a defect on the circle diameter, depending on the curvature of the surface, on the angle between the slope and the plane, and on the distance between circles (Figure 3).

The error on the radius can be limited by reducing the distance between circles. This error should be small in comparison with the error obtained by a direct measurement of the circle.

The calculations below represent a general case for the circularity or for the estimation of a diameter at a given distance from a datum.

**Figure 4:**
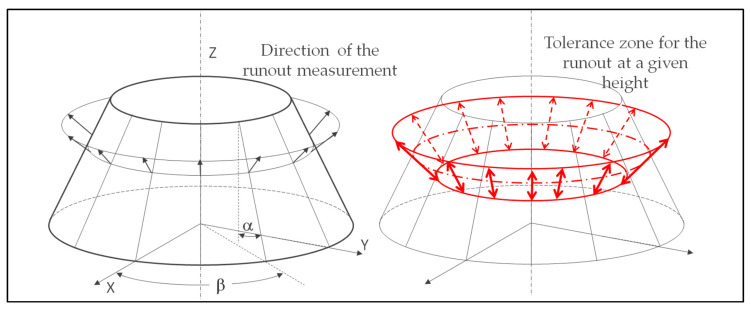
Representation of the direction of the measurement of a runout on a conical surface without any direction feature (black arrows) [7]. Example of tolerance zone for the runout at a given height (red arrows).

The case of the runout is a little different because the part’s defects are measured along a normal vector to the surface (Figure 4).

Equations (1)–(6) remain unchanged, but the intersection with a plane differs because the tolerance zone is conical. This means that the locally constructed plane must be perpendicular to the tangent plane at the theoretical target point.

In this instance, the line constructed from the contact points must be intersected with a plane containing the theoretical coordinates of a point and the vector normal to the surface at that point. There are as many planes as constructed lines. The direction of the measured deviation must follow the theoretical normal vector [7], which these constructions allow.

On a cone with a half angle *α*, this local plane through a point *Pt_i_ (x_i_*, *y_i_*, *z_i_)* belonging to the right cross section can be written thus (*z_i_* is the height of the desired section) (8).

(8) $$ax_{i}+by_{i}+cz_{i}+d=0$$

This includes the following conical relations (9).

(9) $$\left\{\begin{array}{c} x_{i}=r_{i}cos\beta \\ y_{i}=r_{i}sin\beta \\ r_{i}=z_{i}tan\alpha \end{array}\right.$$

The vector of the plane comes from the double cross product between the normal vector and the cone axis (here, *z* axis—(10)).

(10) $$\vec{P_{i}}=\left(\frac{\vec{n_{i}}\wedge \vec{z}}{\left\|\vec{n_{i}}\wedge \vec{z}\right\|}\right)\wedge \vec{n_{i}}$$

So, the plane is defined as follows (11).

(11) $$\left\{\begin{array}{c} a=\vec{P_{i}}.\vec{x}\\ b=\vec{P_{i}}.\vec{y}\\ \begin{array}{c} c=\vec{P_{i}}.\vec{z}\\ d=-ax_{i}-by_{i}-cz_{i} \end{array} \end{array}\right.$$

Then, Equations (5)–(7) can be used, replacing the vector $\vec{axis}$ by the vector $\vec{P}$.

This method can be used to measure a few circles, for example, to define a circle as a secondary datum. However, should there be many circles, the following method is recommended.

### 2.3. Second Method

**Figure 5:**
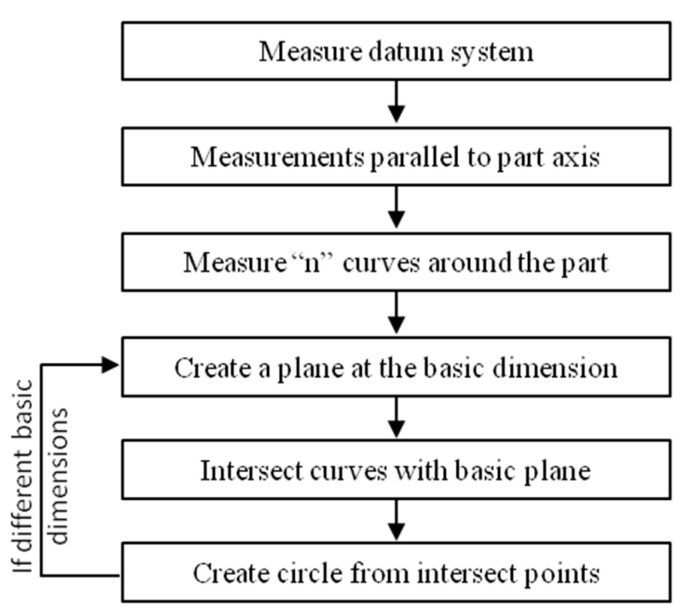
Sequence of operations to perform the second method.

The second solution to avoid the servo-control defect consists of measuring a set of curves around the part, and then intersecting them with a plane to construct a circle (Figure 5).

The main difference between measuring along the curvature or perpendicularly to it is the distance from the center ball to the contact point along the vector of the plane motion. When the plane motion passes through the part’s axis, this distance is very short. This is also the case while measuring a circle perpendicularly to the curvature.

This second method allows many circles to be constructed by the intersection with different planes, answering the ACS specification [7], but it needs scanning capabilities to be performed. The point density of the scan should be matched to the curvature in order to maintain sufficient accuracy. On a curved surface, a density from 10 to 20 points per millimeter should suffice, while a density of 4 points per millimeter on a cone may be appropriate.

### 2.4. Methods Applied to Two Geometrical Configurations

#### 2.4.1. Application to a Calibration Sphere

**Figure 6:**
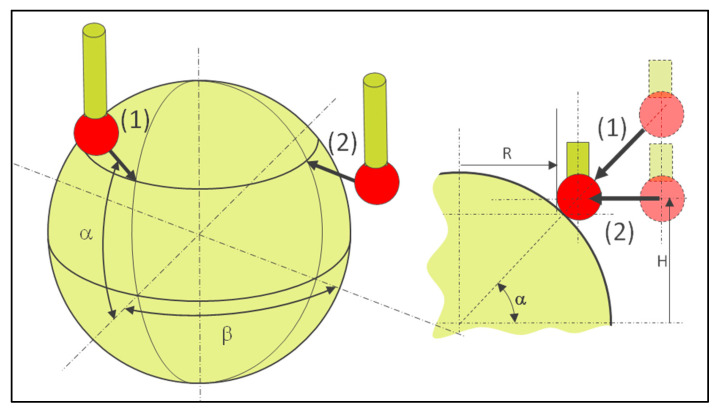
Different directions of probing along the normal vector using vector points (1) or on a plane using a circle program (2); calculation of coordinates to obtain the correct contact point while using a circle program (2).

In order for the method to be demonstrated, it needs to be applied to known features, such as standard parts. The method can be used on a calibrated sphere (Figure 6) at different angles with a gap between circles to minimize the defects. The measured circle is constructed from vector points with a direction perpendicular to the tangent at the target coordinates. In the case of the sphere, the vector is radial.

The coordinates of the target point and the approach vector are calculated as follows (12).

(12) $$\left(\begin{array}{c} \begin{array}{c} x\\ y \end{array}\\ \begin{array}{c} z\\ i \end{array}\\ \begin{array}{c} j\\ k \end{array} \end{array}\right)=\left(\begin{array}{c} \begin{array}{c} r.cos\alpha .cos\beta \\ r.cos\alpha .sin\beta \end{array}\\ \begin{array}{c} r.sin\alpha \\ cos\alpha .cos\beta \end{array}\\ \begin{array}{c} cos\alpha .sin\beta \\ sin\alpha \end{array} \end{array}\right)$$

If circle programming is used, the target coordinates have to be calculated considering the theoretical contact point (see Figure 6).

The coordinates (*H*, *R*) of the contact point are calculated as follows.

(13) $$H={(r}_{sph}+r_{ball})sin\alpha$$

(14) $$R={(r}_{sph}+r_{ball})cos\alpha -r_{ball}$$

**Figure 7:**
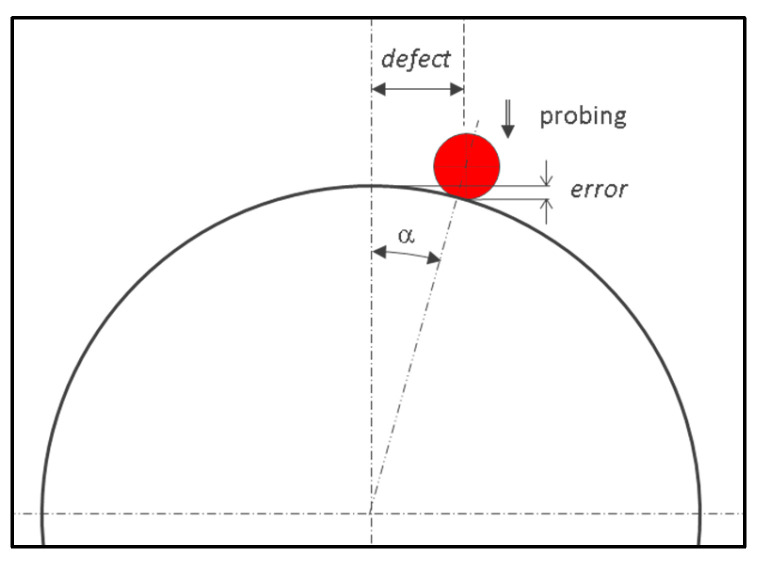
Determination of measurement error while measuring the sphere along a plane passing theoretically through the center using Equations (15) and (16).

If the measurement is carried out at the equator, the defect is negligible (Figure 7).

(15) $$\alpha =atan\left(\frac{defect}{R_{sph}+r_{ball}}\right)$$

(16) $$error=R_{sph}-\left(\left(R_{sph}+r_{ball}\right)cos\alpha -r_{ball}\right)$$

#### 2.4.2. Application to a Torus

**Figure 8:**
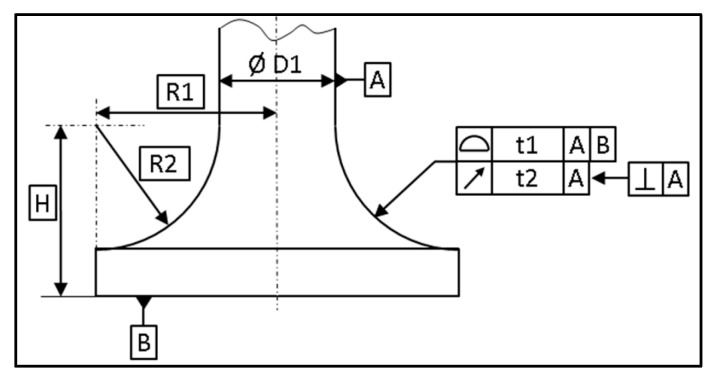
Example of dimensioning for a torus.

A torus is a warped surface with two owned theoretical dimensions, R1 and R2 (Figure 8).

**Figure 9:**
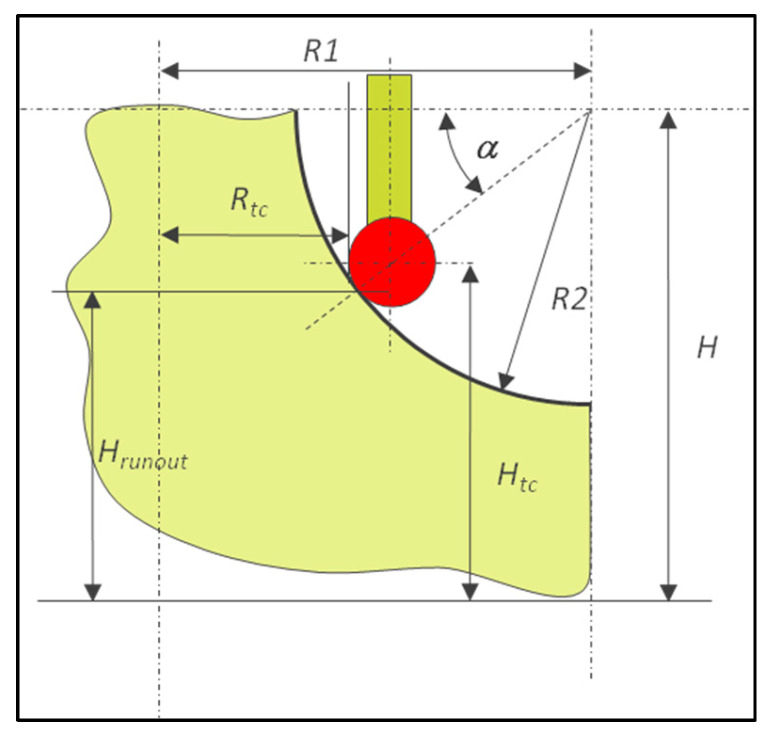
Calculation of different targets to measure the runout on a torus.

**Figure 10:**
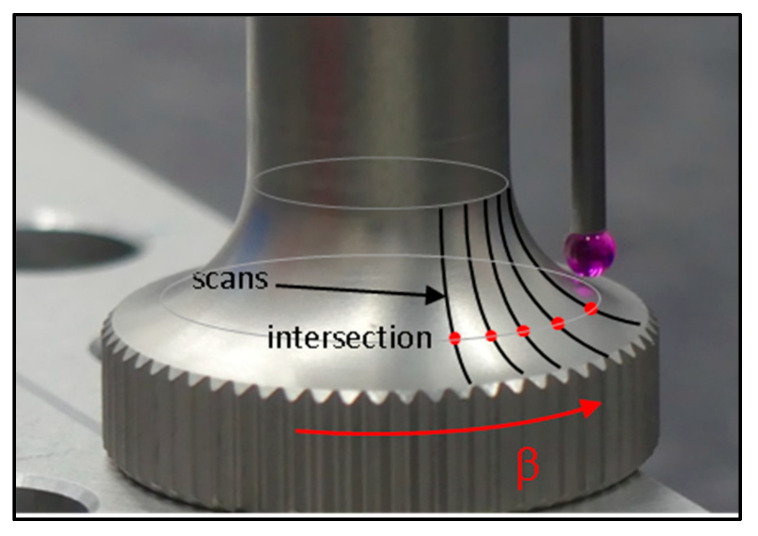
Intersection between a set of scans and a plane at the runout height.

The following methods to measure the runout are applied:

- Direct measurement with vector points perpendicular to the surface (Figure 9),
- Circle constructed from circle hits (Figure 9—first method),
- Scanning the radius R2 at different angles around the part and intersecting them with a plane (Figure 10).

The choice of method depends on various parameters, particularly the size of the part, the number of dimensions to be measured (ACS or not), and the type of probe.

Direct measurement at a given height requires the calculation of the vector direction using (17) and (18).

(17) $$\alpha =\sin^{-1}\left(\frac{H-H_{runout}}{R2}\right)$$

(18) $$\left(\begin{array}{c} \begin{array}{c} x\\ y \end{array}\\ \begin{array}{c} z\\ i \end{array}\\ \begin{array}{c} j\\ k \end{array} \end{array}\right)=\left(\begin{array}{c} \begin{array}{c} \left(R1-R2.cos\alpha \right).cos\beta \\ \left(R1-R2.cos\alpha \right).sin\beta \end{array}\\ \begin{array}{c} H_{runout}\\ cos\alpha .cos\beta \end{array}\\ \begin{array}{c} cos\alpha .sin\beta \\ sin\alpha \end{array} \end{array}\right)$$

The target dimensions for the constructed circle method can be calculated using (17) to define α, replacing *H_runout_* by *H_runout_* ± *d*, with *d* being the distance around *H* (Figure 9).

Then, the targets for circle hits are determined as follows.

(19) $$\left(\begin{array}{c} \begin{array}{c} x\\ y \end{array}\\ \begin{array}{c} z\\ i \end{array}\\ \begin{array}{c} j\\ k \end{array} \end{array}\right)=\left(\begin{array}{c} \begin{array}{c} \left(R1-R_{ball}-\left(R2-R_{ball}\right).cos\alpha \right).cos\beta \\ \left(R1-R_{ball}-\left(R2-R_{ball}\right).cos\alpha \right).sin\beta \end{array}\\ \begin{array}{c} H_{runout}+R_{ball}.sin\alpha \\ cos\beta \end{array}\\ \begin{array}{c} sin\beta \\ 0 \end{array} \end{array}\right)$$

The accuracy for this scanning method depends on the number of hits per millimeter during the scan because the intersection of the scan and the plane to dimension the runout can be a linear interpolation depending on the software.

### 2.5. Part Defects

**Figure 11:**
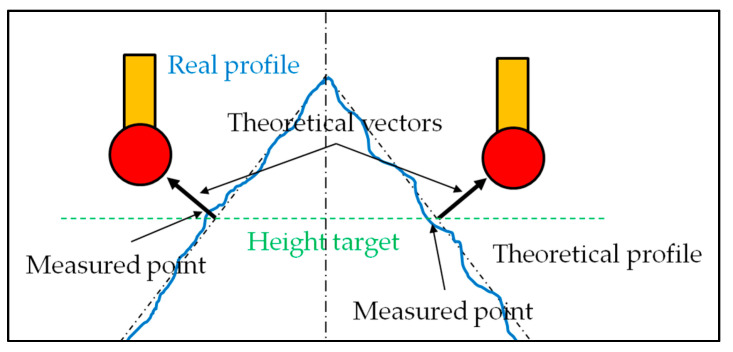
Similarity between servo-control and part faults.

The part itself may present defects equivalent to those created by the servo-control fault, such as roughness or form deviations (Figure 11). In such cases, the proposed methods can be applied in the same way, thus improving the reliability of measurement results.

## 3. Results and Discussion

The results presented in this section are illustrative only. Each user should perform the different tests in order to characterize their CMM. The results have been expressed in the least-squares sense, but it should be noted that any other algorithm is capable of handling point coordinates.

### 3.1. Measurement of Servo-Control Defects

Certain values of servo-control defect on a CMM can be defined by measuring known and calibrated features and looking at hidden parameters, such as the deviations around the target and the form of constructed features.

#### 3.1.1. Case of the Coordinates of a Point

**Figure 12:**
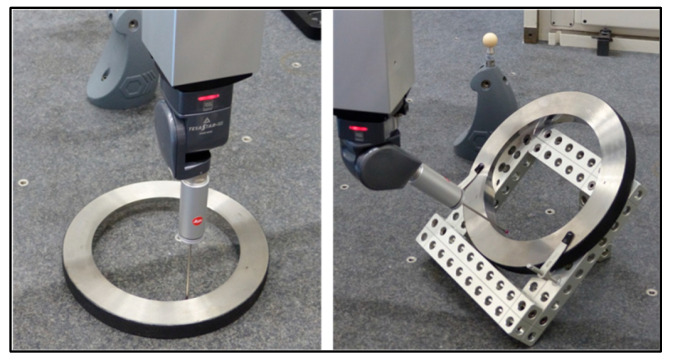
Different positions of the ring gauge in order to characterize the servo-control fault.

We propose to measure the defects on the coordinates of a point. This can be performed on any feature with a known surface defect (flatness, roundness, and so on). We choose to use some ring gauges, which will be used in a second stage. The ring gauges are positioned in two orientations: parallel to the granite and at 45° of each axis (Figure 12).

**Figure 13:**
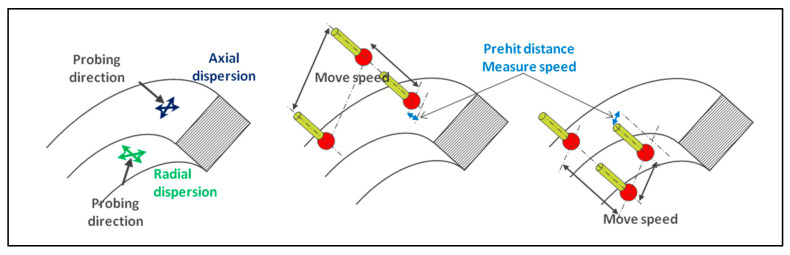
Different dispersions created by a servo-control defect with lateral moves.

The first parameters to be determined are the perpendicular deviations to the tip axis when the probing direction is parallel to it (axial dispersion) and the deviations when the probing direction is perpendicular to the tip axis (radial dispersion—Figure 13).

The theoretical coordinates of the measured point are sent to the CMM through the controller one hundred times in a loop. To maximize the defect, it is important to insert a lateral move point inside looping hits so that the probe moves to a different location, activating different axes and, therefore, different motors, at different speeds (Figure 13).

The test produces different values of dispersion, qualifying the servo-control defects (Table 1). Testing is conducted with the same ring gauge and the same measurement parameters on two different CMMs. Each configuration is run one hundred times, with the results presented as the average deviation from the target and the standard deviation. Table 1 shows a large difference between both CMMs, especially on average defects. CMM1 has an old controller and CMM2 a new one. In addition, on CMM1, the standard deviation of defects is small, showing that the defect has good precision and poor accuracy [28].

#### 3.1.2. Case of a Circle Measurement

This test consists of measuring the circle of the ring gauge in different positions.

First, it is important to level the ring gauge on its top plane, even if it is not the plane of the calibrated diameter, in order to impose the move direction. Depending on the CMM equipment, this test can be conducted point-to-point or by scanning.

**Figure 14:**
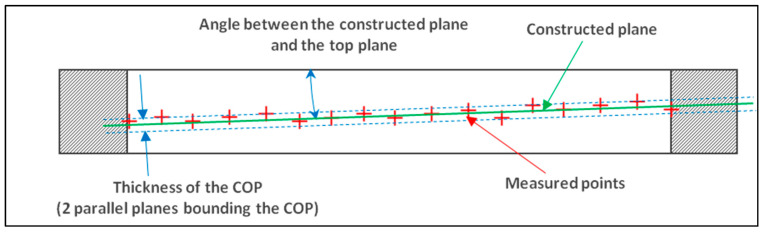
Representation of defects on a measured circle inside a ring gauge: thickness of the cloud of points (COP) and angle between the constructed plane and the theoretical target.

**Figure 15:**
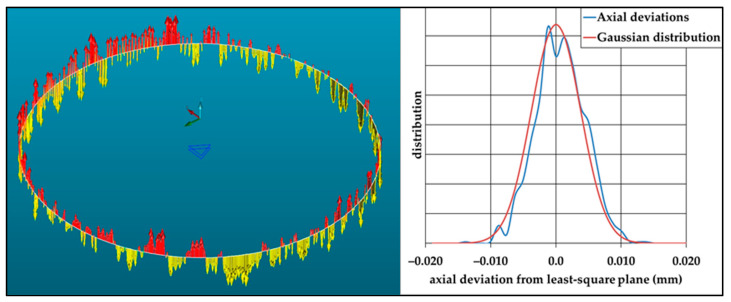
Example of axial dispersion during circle measurement by scanning a ring gauge (Ø 90 mm). The arrows illustrate the distance between the measured points and the constructed plane. The colour of the arrows indicates the sign of the deviation. Comparison with a Gaussian distribution.

The aim of this test is to dimension the dispersion of circle hits along the top plane vector and to dimension the angle between a least-square plane constructed from circle hits and the top plane (Figure 14 and Figure 15).

**Figure 16:**
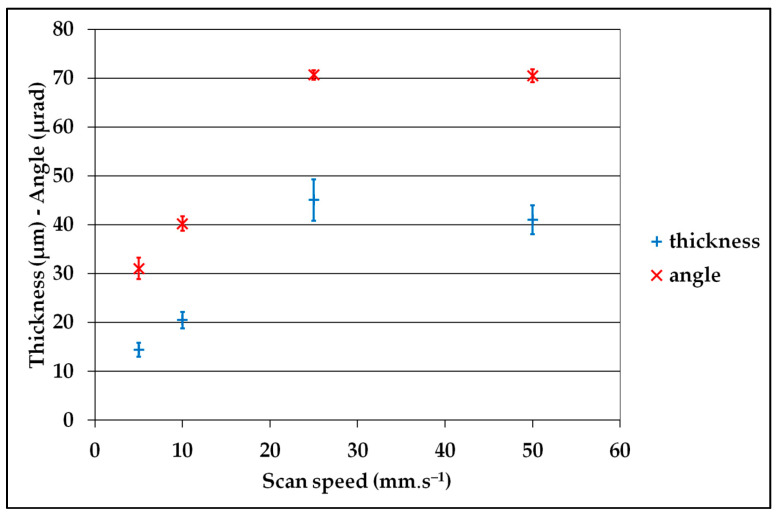
Example of the influence of the scan speed on the thickness and the angle of the plane constructed from the circle hits.

**Figure 17:**
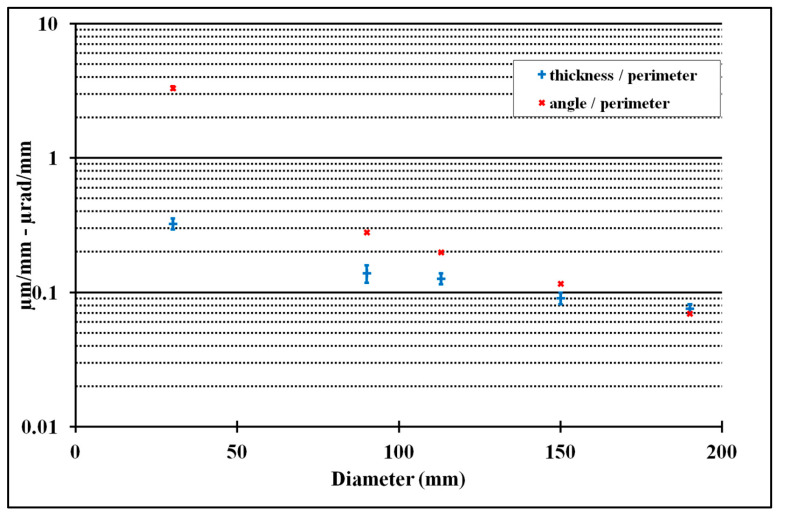
Example of the influence of the measured diameter on the thickness and the angle of the plane constructed from the circle hits.

These defects depend on different parameters, such as the CMM, the position on the CMM, the diameter to be measured, the measurement speed, the type of probe (scanning head or touch trigger probe), the orientation (Table 2), and so on. It is imperative that this test is carried out on dimensions that are close to those usually measured, using standard measurement parameters. (Figure 16 and Figure 17).

The point-to-point circle is measured with 36 points, and the scanned circle with 1130 points. On the CMM used, the defect does not seem to be random. Part of the result is repeatable for the location of the ring gauge, and a small part of the result is random, as shown by standard deviation values.

From one location to another, the average values remain of the same order. For example, on a Ø 113 mm ring measured at two different angles (±45° around the *Z* axis) in two different locations with the same parameters, the results show some differences, confirming that the defect is repeatable but not accurate (Table 3).

### 3.2. Applications to Avoid Servo-Control Defects

In this section, some results are presented, showing the difference between traditional direct measurement and both methods explained above.

#### 3.2.1. Case of a Warped Surface Measurement

**Figure 18:**
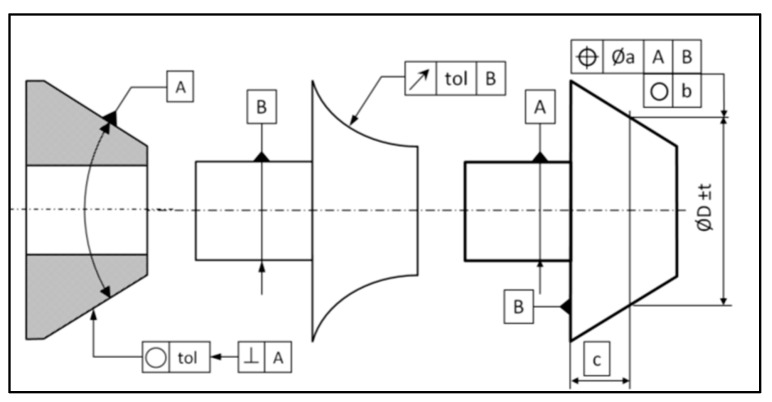
Dimension, form, and location examples impacted by the servo-control defect [7].

Warped surfaces can be measured in different ways to obtain different types of dimensions, such as size or geometrical. On a conical surface, the dimensioning usually concerns the angle and the surface profile, which are 3D parameters. They can be estimated easily, using traditional rules of measurement, such as a sufficient number of hits and a suitable ball radius. On these kinds of axisymmetric surfaces, the dimensioning of 2D parameters can be required, such as a diameter at a basic distance, the circularity, or the runout, as shown in Figure 18.

**Figure 19:**
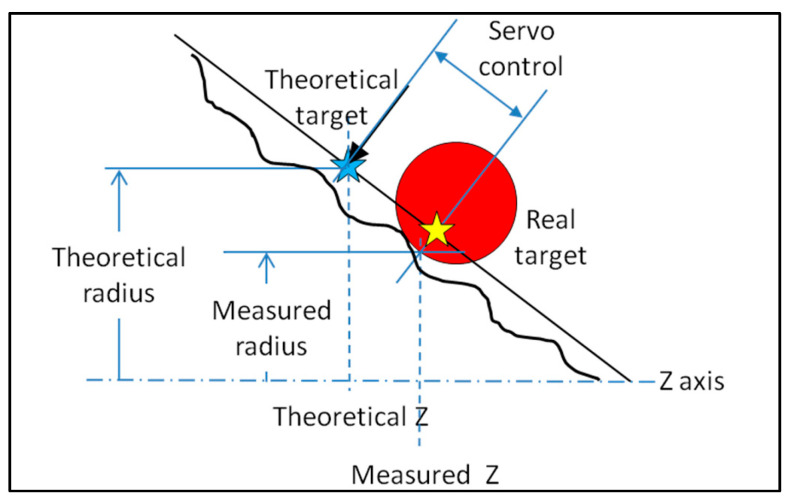
Difference between the theoretical target and the measured coordinates.

Typically, form dimensions on a single surface are applied to any cross sections (ACS [7]) by default. In this case, the measurement should be taken along a perfect plane perpendicular to the part’s axis. The servo-control does not permit accurate execution due to the discrepancy between the theoretical target and the actual one (Figure 19).

**Figure 20:**
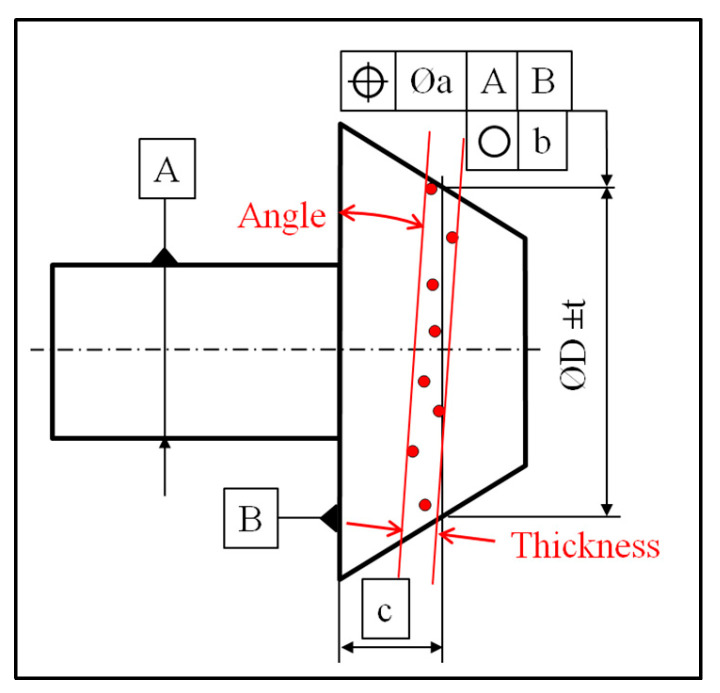
Dispersion of hits (red dots) around a cone due to the servo-control defect.

The direct measurement of a circle on a conical surface is subject to servo-control defects, and the result shows errors on the diameter and the circularity values and on the location of the center (Figure 20).

#### 3.2.2. Demonstration of the Effect on a Virtual Cone

**Figure 21:**
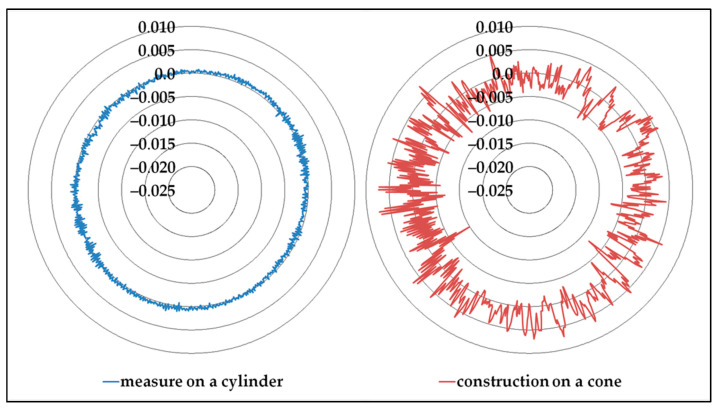
Application of the axial deviations measured on a cylinder to a virtual cone (α = 45°).

The main difference between measuring a circle on a cylinder and measuring a circle on a cone can be demonstrated by a calculation. It is necessary to use the coordinates (*x*, *y*, *z*) of points measured on a cylinder. The coordinates used are expressed relative to the center of the least-squares circle. This set of coordinates can be used to create a circle, and the axial deviations can be used to calculate a radial deviation on a virtual cone, setting its angle (Figure 21).

We used a set of more than 1000 points, measured on a ring gauge by scanning.

The axial deviation of the point *i* is calculated as the difference between the actual *z* value and the average of all *z* values (20).

(20) $$\delta z_{i}=z_{i}-\bar{z}$$

The radial deviation of the point *i* can be estimated as follows, depending on α, the half angle of the cone (21).

(21) $$r_{i}=\sqrt{x_{i}^{2}+y_{i}^{2}}-\delta z_{i}cos(\alpha )$$

The application of axial deviations of the circle measured on a cylinder to a virtual cone demonstrates the effect of servo-control on oblique surfaces. The methods described in this paper allow such considerable differences to be avoided, especially regarding circularity (Table 4).

Using measured coordinates from [24] enables a cone to be constructed. This cone is measured with six different levels of 12 points each. The gap between levels of measure is about 6 mm. To compare different methods, only three levels need to be selected, with the middle one being considered as a direct measurement, and the others as circles around it (first method). The heights selected are 3 mm, −3 mm, and −9 mm, respectively. The profile of the cone constructed from those 36 points is 7 µm. The different results presented in Table 5 relate to:

- A: the measured circle constructed from points on the middle level,
- B: the circle constructed from lines between points on both other levels, using Equations (5)–(7) (coordinates of points are considered as being compensated—first method), with the basic plane at the same height as the middle level, and
- C: the circle constructed from the intersection between the cone and the plane, which represents the intersection of a perfect cone associated with the points in the plane.

Table 5 shows few differences on the diameter because the profile defect of the cone is negligible. However, the coordinates of the center show a greater difference.

It is also possible to construct a plane from points of the direct measurement, and then use this instead of the basic plane in the first method. The center of the circle obtained this way is very close to the center of the direct measurement (*x* = −6 µm and *y* = −4 µm). This confirms the soundness of the effect of servo-control.

#### 3.2.3. Case of a Calibrated Sphere

**Figure 22:**
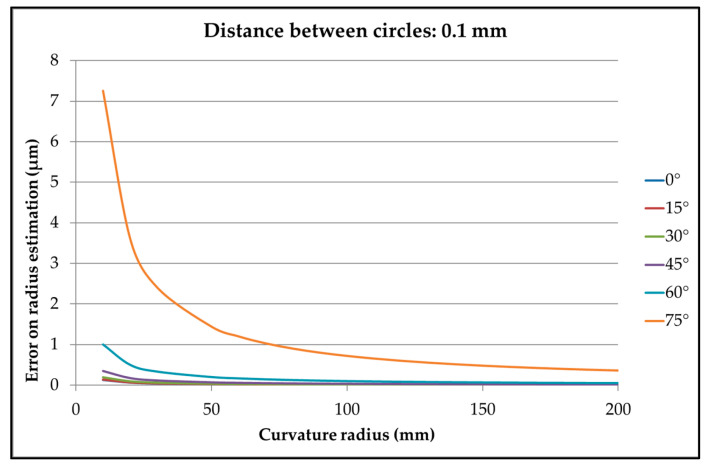
Example of defect on diameter depending on the surface curvature and the angle between the constructed line and the basic plane.

The radius defect described in Figure 3 can be estimated by calculation. An example is shown in Figure 22 for a distance between planes of 0.1 mm (±0.05 mm around the basic plane). There is no radius defect on conical surfaces.

We tested the method on a calibrated sphere at different angles with a gap between circles of 0.04 mm to minimize the defects described above. The constructed circle uses hits of two circles around the basic plane. Equations (13) and (14) are used to calculate the correct targets, and then (1)–(7) are used to calculate the constructed circle.

For example, on a calibrated sphere (Ø 24.9928 mm), a gauge plane is defined at 8 mm from the center, and the probe diameter is 2.999 mm. The calibration uncertainty of the sphere is ±0.3 µm for the diameter and less than 0.08 µm for the roundness.

The first circle’s theoretical target has to be set at 8.9375 mm and the second at 8.9824 mm, so the contact points between the probe and the sphere are at ±0.02 mm around the gauge plane using (13) and (14).

The theoretical diameter is 19.2000 mm, while the constructed diameter is 19.201 mm.

Table 6 shows that the circle constructed using the first method has a roundness close to what is expected for this type of measurement on a calibrated sphere.

In addition, the uncertainty of the diameter estimated by a virtual machine [29] is ±2.8 µm (k = 2) and ±0.7 µm for the roundness. These values show a good correlation compared to the standard uncertainties.

The comparison between direct measurement using the normal direction of probing ((1) on Figure 6) and construction using the first method produces the following results (Table 7). The results do not show a real difference on the location of the center (*x*, *y*) or the circularity, but give clear differences on the *z* value of the center, on the diameter, on the thickness, and on the runout.

Using the coordinates of sph3.ds [25] to construct a circle at 150 mm from the center of the constructed sphere gives the results presented in Table 8. The sphere presents a profile defect close to 0.7 mm. Even if the cloud of points does not come from a real measurement, the treatment via two different methods generates a large difference in results. The aim of this test is to identify a way to dimension the parameters of a circle correctly.

These different measurements of circles on a sphere can be summed up as follows:

- Direct measurement generates errors on diameter, center, and circularity because of the servo-control faults.
- Intersection between a plane and a sphere does not allow access to the circularity and gives faulty results on the location of the center.
- Using the first method gives rise to values meeting the standard specifications in terms of localization, orientation, and thickness. The associated uncertainty may be estimated with a virtual CMM.

#### 3.2.4. Case of a Torus

**Figure 23:**
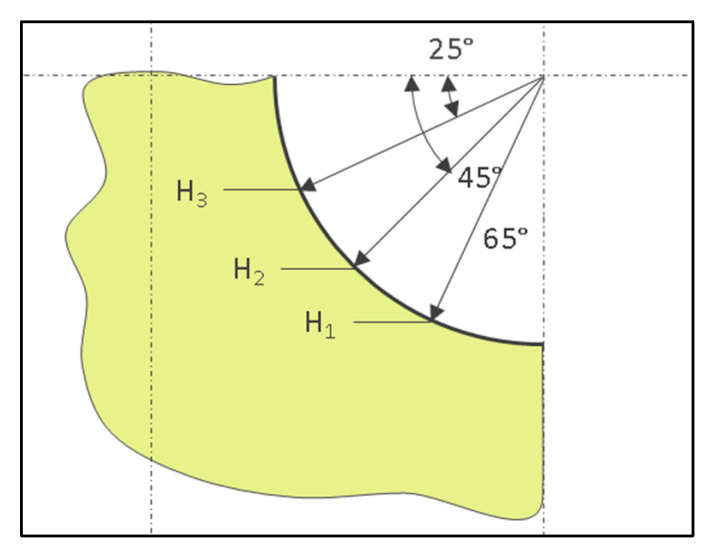
Representation of the different heights where the dimensions are measured.

For the three different methods presented in Section 2, the results are compared for the roundness and the runout at three different heights (Figure 23). We also add the result of the flatness of the plane constructed from direct hits and the angle between this plane and the datum, which should be exactly 90° (Table 9 and Table 10).

**Figure 24:**
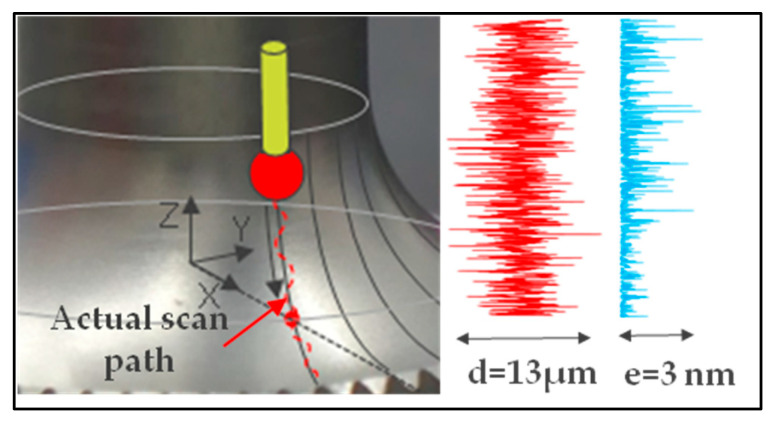
Error *e* on the radius value of a servo-control defect *d* in the case of the torus.

The scan path in a theoretical direction parallel to the revolution axis is subject to the servo-control defect in the same manner as for a measurement of the equator on a sphere (Figure 24).

Table 9 shows that the difference of roundness depending on the method is not significant, but Table 10 shows that the runout presents very significant variations for H_1_ and H_2_ cases.

The direct measurement gives low deviations because the path of the probe is programmed in the datum alignment, which is centered on the cylinder. The polar radius of each hit is imposed, even if the torus is not centered. In this case, it is important to check the thickness and the angle of the plane constructed from hits (Table 10).

Both of the other methods construct a circle by an intersection with a plane at the right height and the right orientation, increasing the accuracy.

With a runout tolerance of 0.1 mm on any cross section (ACS), this part would be accepted through direct measurement, but rejected using the other two methods [7]. In addition, the measured characteristic does not meet the normative specifications for orientation, which is a problem.

The scanning solution gives results close to the constructed circle method and should be the best solution on warped surfaces if the point density is high.

#### 3.2.5. Other Applications

The servo-control defect is often negligible and overlooked. However, it can be evaluated from data, assuming that the measurements are carried out on cylindrical surfaces. Using data of cir1.ds and cir32.ds from NIST [25] allows for the evaluation of the thickness of the measured points and an angle defect for the first file, assuming the vector of the plane is theoretically along the *Z*-axis. The circle cir32 is inclined by about 54° from the vertical, and there is no information about the theoretical angle.

Values from [23] can also be used to estimate the thickness of the set of points and the angle of the constructed plane.

It is also possible to do this with the coordinates from [24], measured on a cone, but they take into account the part’s defect in addition to the lack of servo-control. These coordinates represent six circles measured on a cone at different heights; the results presented are the average of thickness and angle (Table 11).

Using measured coordinates from [24] allows for the construction of a cone. This cone has a profile defect of above 0.1 mm. The gap between levels of measure is about 6 mm. The alignment is constructed along the cone axis.

The construction of a diameter on a gauge plane between two levels, as opposed to the utilisation of the described method, yields a substantial difference (Table 12). The result of the construction of a circle at a gauge plane is the intersection between the plane and the associated perfect surface, which depends on the algorithm used. This circle has no profile defect by definition and is centered on the axis of the surface. Constructing the circle by intersection “point-to-point” enables the evaluation of the circularity and a real center.

Some CMM software applications allow this kind of defect to be corrected on spherical or conical surfaces by projecting real hits along the surface associated with the basic plane. This kind of filter does not work on warped surfaces because of a lack of mathematical definition of the surface. In addition, the part itself causes defects.

Without any correction, the measured circle presents incorrect values on its diameter, its center, and its form defect, i.e., on all the most interesting dimensions of a circle. Whenever an axial deviation of the probe leads to a radial deviation, a correction must be applied. The uncertainty of the correction can be evaluated using [29].

## 4. Conclusions

The use of a CMM facilitates a range of capabilities while simultaneously minimizing the number of expensive machines and the frequency of part displacements between different machines. In this article, we focus on a specific parameter, servo-control, which is not often discussed in the context of CMMs, but rather in that of machine tools. Although this functionality of CMMs rarely leads to significant defects, defects might be introduced to certain dimensions of measured elements. In such cases, it is necessary to construct the elements to be dimensioned, rather than measuring them directly, in order to ensure the highest possible accuracy. The issue arises with roundness and runout, particularly when the latter is dimensioned from a measurement taken on a warped surface. It is important to be aware that direct measurement can lead to the introduction of defects, such as those affecting the diameter, center, and roundness of a circle, which are the only relevant parameters. Furthermore, the results obtained from the direct measurement strategy do not align with the established standard specifications regarding orientation and localization. In sum, the direct measurement method is notable for its simplicity and speed; yet, in some cases, particularly on warped surfaces, the results are not always accurate.

This paper provides an explanation for the causes of these defects and puts forward two different solutions to avoid errors in dimensioning. Both methods are based on the construction of circles from the intersection of segments with a perfect plane. The segment can be constructed in two different ways: from the points of two circles or from linear scans. Whilst both of these methods require a greater investment of time than the direct method, they deliver optimal accuracy and meet the required standard specifications.

By presenting several examples drawn from our CMM or the relevant literature, we illustrate the limitations of servo-control and demonstrate the efficacy of the proposed method. Furthermore, we provide a comprehensive explanation of the role of this hidden parameter in measuring circles on left-handed surfaces as opposed to surfaces that are nominally cylindrical.

In certain instances, the errors caused by servo-control can be of a significantly higher order than the typical uncertainty associated with circle measurements. In such cases, the loss of a small amount of time can result in a significant gain in terms of quality, allowing for a well-informed decision to be made regarding product conformity.

The key benefit of the suggested methodologies lies in their versatility, which allows them to adapt to multiple algorithms, in particular those related to circular datum. It is imperative to apply this method to a specified secondary or tertiary reference when implementing the datum system to ensure its proper construction. Should the software’s options prove inadequate in facilitating the straightforward construction of the intersection points described, a background or post-processing calculation using the equations provided in this paper can ensure a more reliable result.

Our study was conducted using a bridge coordinate measuring machine (CMM), the most common type of CMM. The suggested tests could also be performed on other types, such as gantry CMMs, cantilever CMMs, and horizontal arms, which may exhibit different behaviors. The same holds true for five-axis scanning heads, TTP, or CMMs equipped with a rotary table. High-speed scanning in the shape of a tight sinusoid around the intersection plane would enable the circle to be created with great efficiency. This could be a valuable research area for software publishers.

It would also be beneficial to locate information regarding servo-control within the CMM documentation. In addition, it would be beneficial to incorporate a chapter on this subject into the ISO 10 360 standards, or their equivalent in the American Society of Mechanical Engineers (ASME).

In sum, regardless of the type of CMM or probe, it is essential to perform these tests for two primary reasons. First, they ensure the correction of servo-control defects. Second, they provide a comprehensive understanding of CMM functionality.

## Figures and Tables

**Table 1:** Probing deviations from two different CMMs.

Probe Orientation	Probing Direction	Target Deviation (µm)/Standard Deviation (µm)	
		CMM 1	CMM 2
vertical	axial	3/0.3	1.4/0.4
vertical	radial	9/0.2	1.1/0.3
angled	axial	18/1.3	1.6/0.4
angled	radial	12/1.4	3/0.4

**Table 2:** Results of the test on the CMM2, with a ring gauge Ø 90 mm measured ten times in each position.

Mode	Probe Orientation	Mean/Standard Deviation	
		Thickness (µm)	Angle (µrad)
point-to-point	vertical	1.2/0.1	4.5/0.9
scanning	vertical	9/1.6	10/1.5
point-to-point	angled	7/0.4	45/3
scanning	angled	39/3	79/0.6

**Table 3:** Results of the test at two different angles.

Mode	Probe Orientation	Mean/Standard Deviation	
		Thickness (µm)	Angle (µrad)
Position 1	Angle1	40/2.2	83/0.6
Position 2	Angle2	45/2.1	71/0.5

**Table 4:** Comparison between a circle measured on a cylinder and its axial deviations applied to a virtual cone.

	Theoretical Value (µm)	Cylinder (µm)	Virtual Cone (µm)
Center (*x*)	0	<10^−4^	0.8
Center (*y*)	0	<10^−3^	1.5
Circularity	0	3.1	17.2

**Table 5:** Comparison between a circle measurement, a constructed circle using the first method described above, and a constructed circle at a gauge plane on a cone.

	A: Direct Measurement	B: Using First Method	C: Construction at a Gauge Plane
Diameter (mm)	38.744	38.746	38.745
Circularity (mm)	0.008	0.004	0
Center (*x*, *y*, *z*) (mm)	−0.006, −0.003, −3	−0.014, −0.013, −3	−0.006, −0.005, −3
Thickness (mm)	0.006	0	0
Angle/*Z* axis (°)	0.24	0	0

**Table 6:** Measuring two circles to construct another one using the first method.

	Roundness (µm)	Thickness (µm)
Circle 1	8.3	11
Circle 2	5.5	8
Constructed circle	1.8	0

**Table 7:** Different results between direct measurement and construction of a circle on a calibrated sphere (36 points).

Parameter	A: Direct Measurement	B: Using First Method	Theoretical Value
Center *x* (µm)	−0.8	−0.5	0
Center *y* (µm)	−0.2	0.2	0
Center *z* (µm)	−10	0	0
Diameter (mm)	17.691	17.672	17.672
Circularity (µm)	4	3	0
Thickness (µm)	4	0	0
Runout (µm)	9	1.1	0

**Table 8:** Comparison between a constructed circle at a gauge plane on a sphere and the first method described above, point-to-point.

	Direct Construction (mm)	First Method (mm)
Diameter	215.315	215.226
Circularity	0	0.587
Center (*x*, *y*, *z*)	0, 150, 0	−0.035, 150, 0.052

**Table 9:** Results of roundness depending on the method and the height, including the defect of thickness of the cloud of measured points.

Roundness (µm) (Thickness of the COP)			
	Direct Measurement	First Method	Second Method (20 pt/mm)
H_1_	8 (4 µm)	4 (0 µm)	6 (0 µm)
H_2_	5 (6 µm)	1 (0 µm)	3 (0 µm)
H_3_	2 (5 µm)	1 (0 µm)	2 (0 µm)

**Table 10:** Results of runout depending on the method and the height, including the defect of angle between the cloud of measured points and the datum.

Runout (µm) (Angle Between the Cut Plane and the Datum)			
	Direct Measurement	First Method	Second Method
H_1_	39 (90.21°)	256 (90°)	257 (90°)
H_2_	71 (90.16°)	115 (90°)	115 (90°)
H_3_	56 (90.1°)	71 (90°)	69 (90°)

**Table 11:** Thickness and angle calculated from coordinates of hits of different circles.

	Thickness (mm)	Angle (°)
Circle [23]	0.0077	0.0005
Circles [24] (average)	0.0068	0.2463
Cir1 [25]	0.0427	0.0094
Cir32 [25]	0.0215	-

**Table 12:** Comparison between a constructed circle at a gauge plane on a cone and the first method described above, point-to-point.

	Direct Construction (mm)	First Method (mm)
Diameter	37.955	37.873
Circularity	0	0.006
Center (*x*, *y*, *z*)	0, 0, 0	0.001, 0, 0

## Data Availability

All the necessary information is provided in this article. For further information, please contact the corresponding author.

## Abbreviations

The following abbreviations are used in this manuscript:

ACS	Any Cross Section
CMM	Coordinate Measuring Machine
CNC	Computer Numerical Control
COP	Cloud Of Points
GPS	Geometrical Product Specifications
ISO	International Organization for Standardization
NIST	National Institute of Standards and Technology
